# Systematic Review on Acupuncture for Treatment of Dysphagia after Stroke

**DOI:** 10.1155/2017/6421852

**Published:** 2017-08-09

**Authors:** Qiuping Ye, Yu Xie, Junheng Shi, Zhenhua Xu, Aihua Ou, Nenggui Xu

**Affiliations:** ^1^Guangzhou University of Chinese Medicine, Airport Road, Baiyun District, Guangdong, Guangzhou 510006, China; ^2^Guangdong Provincial Hospital of Chinese Medicine, Yide Road, Yuexiu District, Guangdong, Guangzhou 510006, China

## Abstract

**Objective:**

To assess the therapeutic efficacy of acupuncture for dysphagia after stroke.

**Methods:**

Seven electronic databases were searched from their inception until 31 September 2016. All randomized controlled trials (RCTs) incorporating acupuncture or acupuncture combined with other interventions for treatment of dysphagia after stroke were enrolled. Then they were extracted and assessed by two independent evaluators. Direct comparisons were conducted in RevMan 5.3.0 software.

**Results:**

6010 patients of 71 papers were included. The pooled analysis of efficacy rate of 58 studies indicated that acupuncture group was superior to the control group with moderate heterogeneity (RR = 1.17, 95% CI: 1.13 1.21, *Z* = 9.08, and *P* < 0.00001); meta-analysis of the studies using blind method showed that the efficacy rate of acupuncture group was 3.01 times that of control group with no heterogeneity (RR = 3.01, 95% CI: 1.95 4.65, *Z* = 4.97, and *P* < 0.00001). Only 13 studies mentioned the safety evaluation.

**Conclusion:**

The result showed that the acupuncture group was better than control group in terms of efficacy rate of dysphagia after stroke. And the combining result of those researches using blind method was more strong in proof. Strict evaluation standard and high-quality RCT design are necessary for further exploration.

## 1. Introduction

Dysphagia was one of the most common sequelae after stroke. The incidence reached 81% [1]. There were many complications in dysphagia, such as cacotrophy [2], dehydration, aspiration, and pneumonia [3]. Those complications improve the morbidity, mortality, the rehabilitation, and the quality of life of the patients. So the medication and intervention time are very important for recovery. Acupuncture was an effective method and more and more welcomed and applied clinically [4]. There were many studies [5–7] about the acupuncture for treatment of dysphagia after stroke internationally, including the scalp acupuncture, nape needle, auricular needling, or combing with other methods.

Though, there were some systematic reviews focusing on the acupuncture for treatment of dysphagia in stroke. There was lack of higher quality research or the positive conclusion could not be obtained. Thus, the inclusion and exclusion criteria were formulated after integrating the previous relevant reports. And the studies using single blind method were pooled to be analysed alone.

## 2. Method and Data

The criterion of systematic review was published in the Cochrane Collaboration which was available on http://handbook.cochrane.org/.

### 2.1. Type of Studies

All articles were included that reported an RCT in patients with dysphagia after stoke. And the animal experiments were not inclusive.

### 2.2. Participants

All the patients should conform to the explicit clinical diagnosis criteria of stroke and dysphagia. They should meet the following diagnosis of stroke: (1) the diagnostic criterion of the Fourth National Conference on cerebrovascular diseases in 1994 or the revised diagnostic criterion in 1995 or 1996; (2) the revised “Various Types of Cerebrovascular Disease Diagnosis Points” of The Fourth National Conference on cerebrovascular diseases of Chinese Medicine Association; (3) the “Chinese Cerebrovascular Disease Prevention And Treatment Guidelines (Try Out)” established by Neurology Branch of Chinese Medical Association according to the 2005 or 2007 Disease Control Division; (4) 1996 Chinese Medicine Internal Medicine Association “criterion for evaluating curative effect of apoplexy”; (5) the guidelines for diagnosis and treatment of acute ischemic stroke composed by cerebrovascular branch of Neurology of Chinese Medical Association; (6) the guidelines for diagnosis and treatment of acute ischemic stroke in China 2010 Edition; (7) National Institutes of Health Stroke Scale (NIHSS) [8, 9]; (8) the therapeutic efficacy evaluation standard of TCM diagnosis for stroke; (9) confirmed by head CT or MRI and other imaging methods for stroke; (10) Summary of the Sixth National Conference on cerebrovascular diseases.

### 2.3. Interventions

For the intervention in acupuncture group, acupuncture alone or combined with other interventions was all included, such as the rehabilitation training, swallowing therapeutic apparatus, swallowing training, and electrical stimulation. There was no distinction for the acupuncture manipulation, acupoint, stimulation intensity, and course of treatment. It is available for blank control group, drugs, or rehabilitation training in control group.

### 2.4. Outcome Measurement

The clinical symptoms had obviously improved with specific evaluation criteria such as the (1) Watian Swallowing Test (WST) [10]; (2) Standardized Swallowing Assessment (SSA) [11]; (3) Ichiro Fujishima Rating Scale (IFRS) [12]; (4) Caiteng 7 Rank for dysphagia [13] or with using the objective index as the efficacy evaluation criterion, such as (1) video-fluoroscopic swallowing study [14]; (2) endoscopic evaluation of swallowing [15]; (3) fluorescence barium swallowing radiography score [16] which were recognized as swallowing assessment.

### 2.5. Information Sources and Search Strategy

We search the following electronic databases from their inception until September 30, 2016: Science Citation Index (SCI), Plumbed, The Cochrane Library, EMBASE, Chinese National Knowledge Infrastructure (CNKI), WanFang Database, Chinese Scientific Journals Database (CSJD), and Chinese Biomedical Literature Database (CBM). The searching terms include “stroke”, “apoplexy”, “cerebral hemorrhage”, “acicula”, “acupuncture”, “impaired swallowing”, and “dysphagia”.

### 2.6. Data Extraction

**Figure 1:**
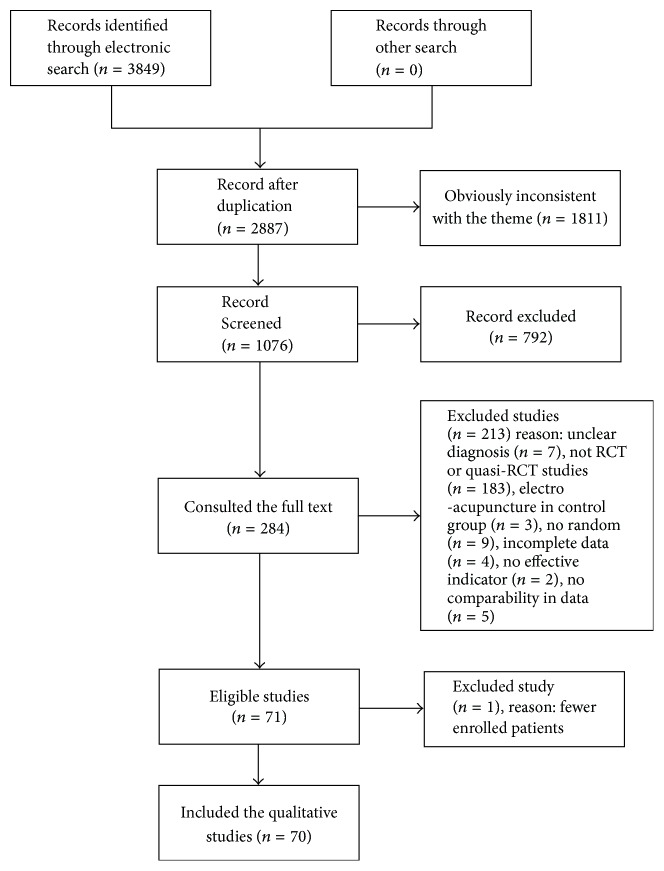
The screening flow diagram.

Data were extracted independently by two authors (Qiuping Ye and Yu Xie) using a specifically designed data extracted form. The disagreements were solved by the third author's assistance (Junheng Shi) if necessary. The following information was extracted: (1) the first author, year of publication and the journal; (2) the research design; (3) the basic situation of the patients; (4) the inclusion and exclusion criteria; (5) the indicators of evaluation; and so on. After recording the reasons for exclusion, we got the flow diagram (see Table 1 and Figure 1) including 71 studies [17–87].

### 2.7. Quality Assessment

**Figure 2:**
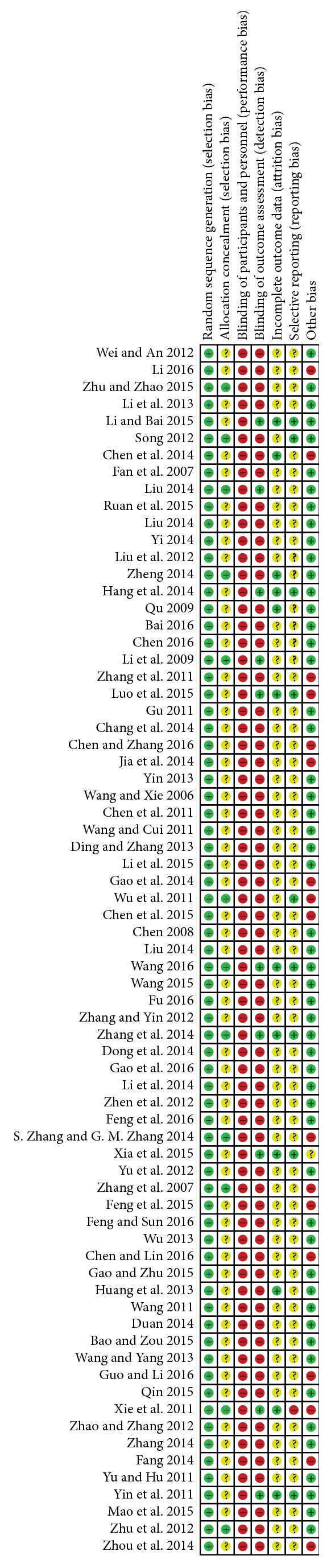
The bias of each study.

**Figure 3:**
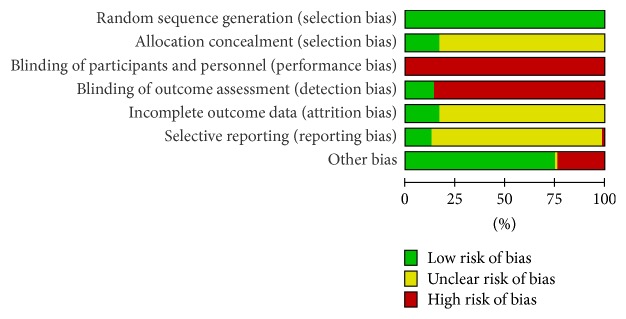
The summary of bias evaluation for the studies.

The methodological quality of each study was assessed from the following aspects: (1) random sequence generation; (2) allocation concealment; (3) blinding of participants and personnel; (4) blinding of outcome assessment; (5) incomplete outcome data; (6) selective reporting; (7) other bias and judging from “yes (low risk),” “no (high risk),” or “unclear (information is insufficient to evaluate)” and reporting the risk of bias graph (Figures 2 and 3).

## 3. Result

71 studies including 6010 patients were enrolled finally. There were 2991 participants in acupuncture group and 3019 participants in control group.

### 3.1. The Basic Characteristics

Two groups were compared statistically based on age, gender, duration, and degree of dysphagia. And the baseline was comparable. See Table 2; 12 studies used the complete random and allocation concealment; 10 studies used the single blind method in the outcome assessment and statistics analysis. For the incomplete outcome data, 12 studies reported the fall off and exit of patients without any effect on the result; 17 studies mentioned the funding support, and not the others.

### 3.2. Data Analysis

RevMan 5.3.0 software was used for data analysis. And the different outcome assessment indicators were used to be classified and analysed. They were presented as risk ratio (RR) or mean difference (MD) with a 95% confidence interval.

### 3.3. Efficacy Rate

**Figure 4:**
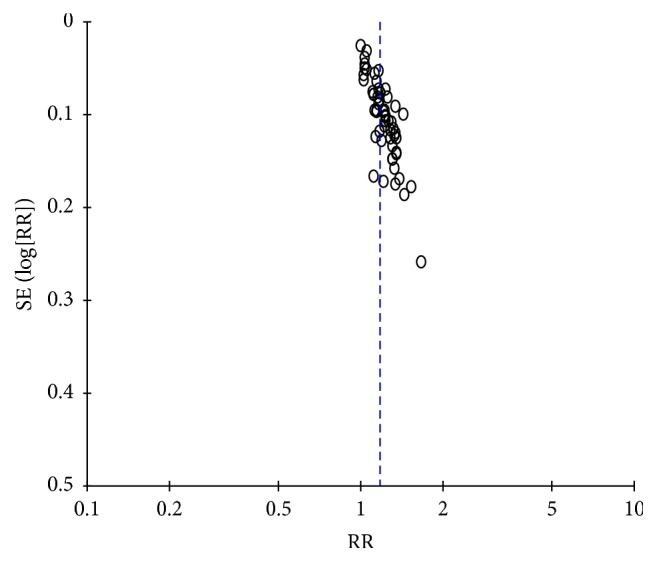
The funnel plot of clinical efficacy rate.

**Figure 5:**
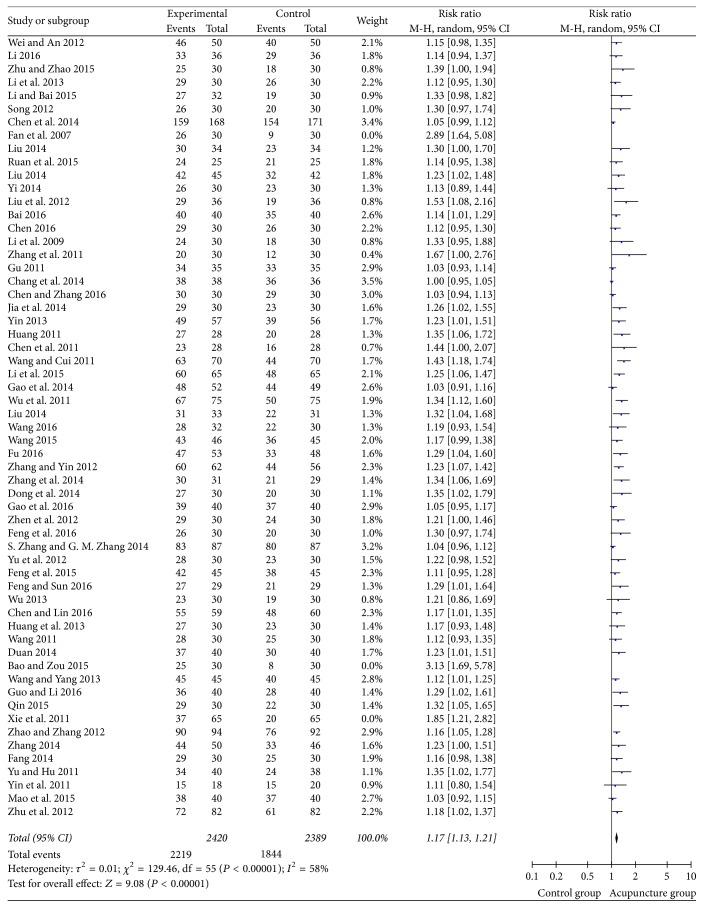
The forest diagram of the clinical efficacy rate.

62 studies used the clinical efficacy rate as the evaluation indicator with the dichotomous data. So the risk ratio (RR) was used to show the result. We found the medium heterogeneity (*I*^2^ = 68%) after combining data. We could observe from the funnel plot that 3 studies [19, 31, 82] had deviated from the center line. After sensitivity analysis, we found that one study [19] considered the significantly effective result as recovery and the other as invalidation, which led to difference in results. At the same time, the intervention group of the two studies [31, 82] was treated with acupuncture combined with western medicine. And the curative effect was significantly higher than that of the control group. All the dots were equally distributed on both sides of the dashed line in the funnel plot with no publication bias after removing them (Figure 4). The moderate heterogeneity was found after remerging (*I*^2^ = 58%). So we chose the random effect model (Figure 5). The pooled analysis showed that the total rectangle was on the right of the equivalent line, which indicated the curative effect of acupuncture group was better than the control group (RR = 1.17, 95% CI: 1.13 1.21, *Z* = 9.08, and *P* < 0.00001).

### 3.4. Standard Swallowing Assessment (SSA)

**Figure 6:**
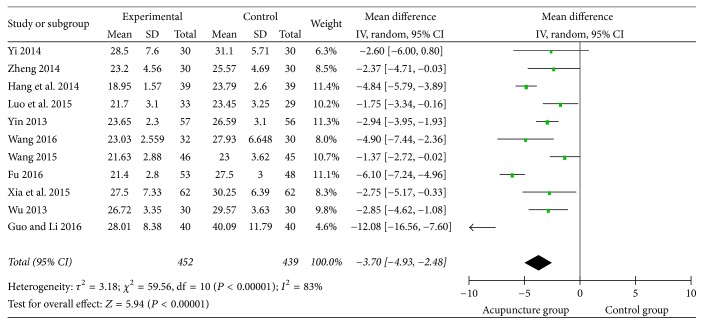
The forest diagram of SSA effective rate.

There were 11 studies that used the SSA as the effective evaluation standard with the continuous data. The meta-analysis of them was showed in mean difference with high heterogeneity (*I*^2^ = 83%). So the random effect model was used (Figure 6). The figure showed that acupuncture group could lower the SSA cores (MD = 3.7, 95% CI: −4.93 −2.48, *Z* = 5.94, and *P* < 0.00001).

### 3.5. Watian Swallowing Test

**Figure 7:**
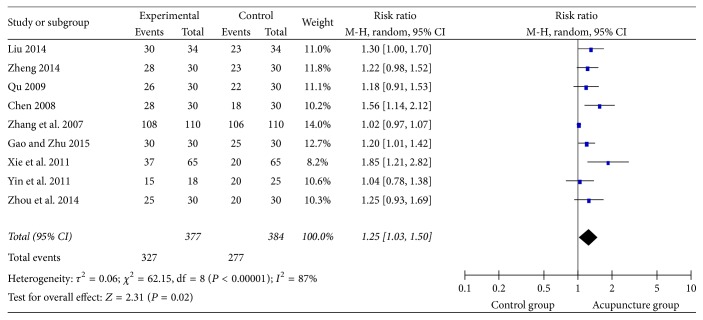
The forest diagram of WST effective rate.

The Watian Swallowing Test was used in 24 studies; 9 of them used the dichotomous data. The risk ratio was selected to demonstrate the count data. The results (Figure 7) showed high heterogeneity (*I*^2^ = 87%). Hence the random effect model was used. And the rectangle was on the right of the equivalent line, which indicated that acupuncture group could improve the efficacy of dysphagia after stroke (RR = 1.25, 95% CI: 1.03 1.50, *Z* = 2.31, and *P* = 0.02 < 0.05).

**Figure 8:**
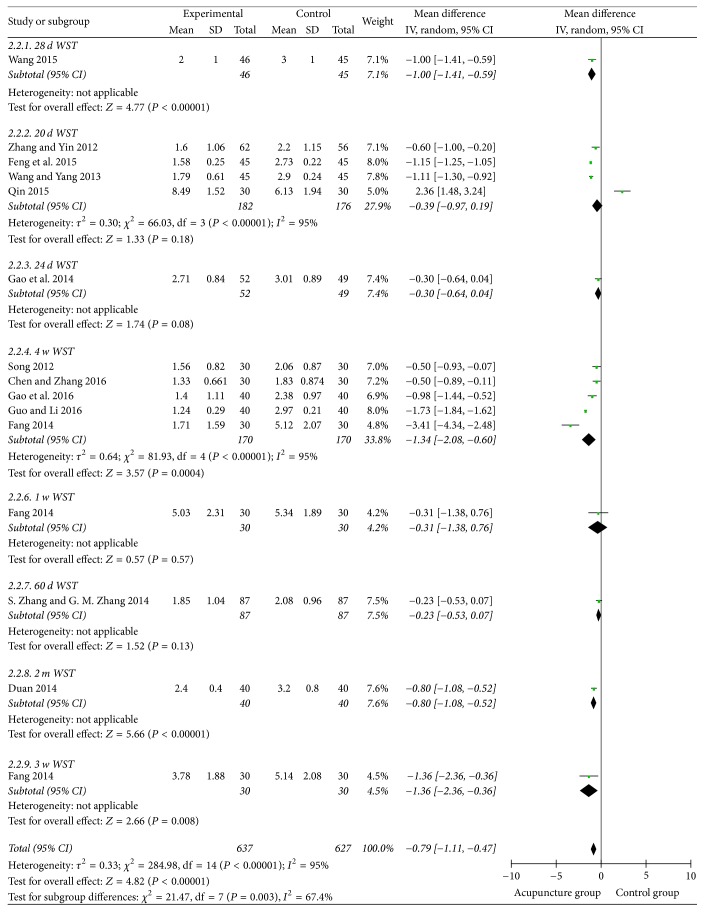
The forest diagram of WST subgroup analysis.

**Figure 9:**
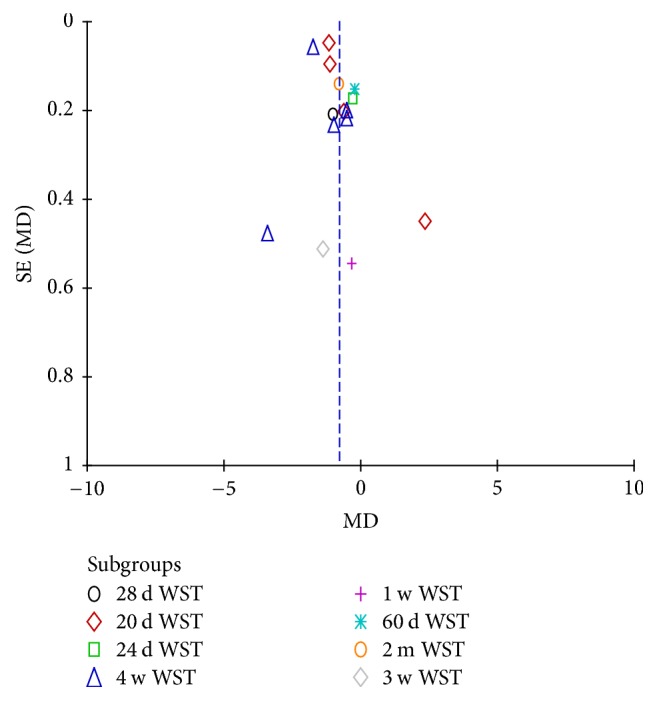
The funnel of WST subgroup analysis.

15 studies used the continuous data. And the mean difference was applied. The results showed that the heterogeneity of the merger was large. So we did the subgroup analysis according to the course of disease. Then the heterogeneity decreased from 95% to 67.4% (Figure 8). There was no publication bias in the funnel plot (Figure 9). Meanwhile, the pooled analysis showed that the acupuncture could lower the Watian Swallowing Test score (MD = 0.97, 95% CI: −1.11 −0.47, *Z* = 4.82, and *P* < 0.00001).

### 3.6. Swallowing Functional Assessment

**Figure 10:**
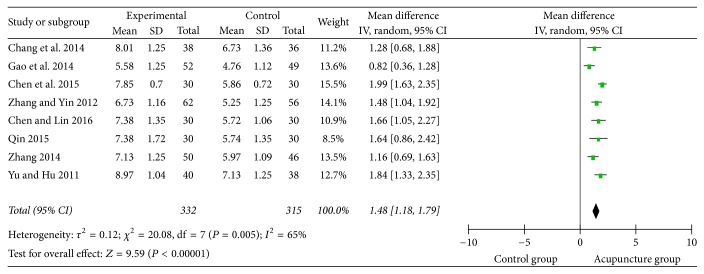
The forest diagram of swallowing function.

Among the included studies, 8 of them used the Swallowing Functional Assessment to evaluate the effectiveness of treatment with the continuous data. The result (Figure 10) exhibited the medium heterogeneity (*I*^2^ = 65%) with mean difference (MD). The result explained that acupuncture could improve the swallowing function with the random effect model (MD = 1.48, 95% CI: 1.18 1.79, *Z* = 9.59, and *P* < 0.0001).

### 3.7. Swallowing Disorder Integral

**Figure 11:**
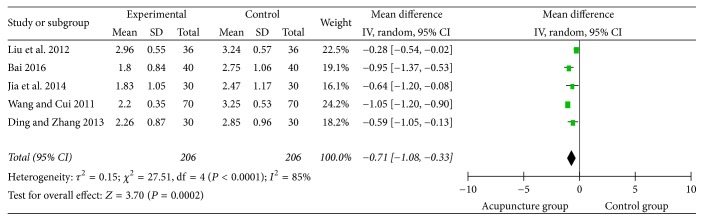
The forest diagram of swallowing disorder integral.

5 studies selected the swallowing disorder scoring as evaluated standard. The meta-analysis of the 5 dichotomous data sets showed that the heterogeneity decreased from 85% to 40% after removing one study [59]. The sensitivity analysis indicated that the heterogeneity might be the treatment course of this study which was longer than the others. We could see from the figure that the score of the control group was higher than the acupuncture group (Figure 11). It illustrated that acupuncture group was able to lower the swallowing disorder integral (MD = −0.71, 95% CI: −1.08 −0.33, *Z* = 3.7, and *P* = 0.0002).

### 3.8. Swallowing-Related Quality of Life (SWAL-QOL)

**Figure 12:**
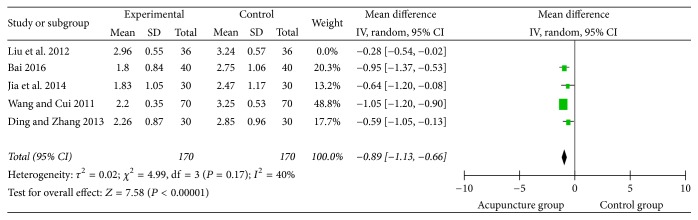
The forest diagram of SWAL-QOL.

5 studies used the SWAL-QOL to express the Swallowing-Related Quality of Life before and after treatment. They all used the continuous data and mean difference to exhibit the results. The pooled analysis showed that rectangle was intersected with the equivalent line with high heterogeneity (*I*^2^ = 100%), which means nothing (Figure 12).

### 3.9. Activities of Daily Living (ADL)

**Figure 13:**
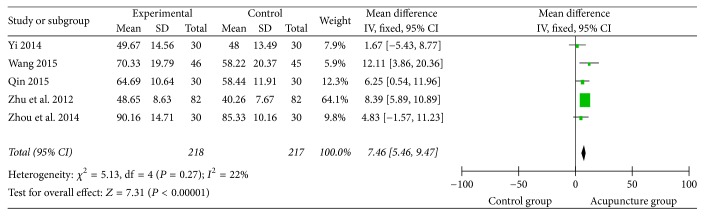
The forest diagram of ADL.

2 studies [67, 78] used ADL to express the curative effect, two [27, 45] used the Barthel index, and the other one [54] used modified Barthel index. Among them, the activities of daily living before and after treatment were showed using the continuous data and mean difference. The meta-analysis indicted that acupuncture group obviously improved the activities of daily living of the patients with lower heterogeneity (*I*^2^ = 22%) (Figure 13). And it was 7.31 times as much as the control group (MD = 7.46, 95% CI: 5.49 9.47, *Z* = 7.31, and *P* < 0.0001).

### 3.10. Caiteng 7 Rank (CT7R)

**Figure 14:**
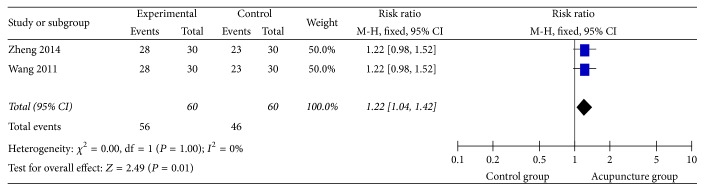
The forest diagram of Caiteng 7 Rank.

The CT7R was used in 2 studies [48, 87] with dichotomous and risk ratio. There was no heterogeneity (*I*^2^ = 0%) after combining the data with the fixed effect model (Figure 14), which indicated that the Caiteng 7 Rank scores of the acupuncture group were higher than the control group (RR = 1.22, 95% CI: 1.04 1.42, *Z* = 2.49, and *P* = 0.01).

**Figure 15:**
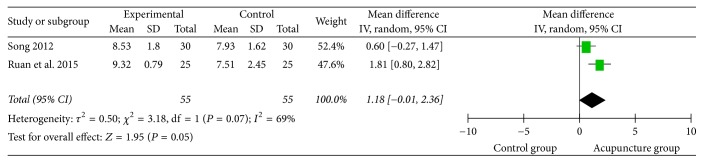
The forest diagram of IFRS.

The pooled analysis (Figure 15) of the 2 studies [44, 56] using Ichiro Fujishima Rating Scale (IFRS) showed no meaning with medium heterogeneity (*I*^2^ = 69%), neither the result of 2 studies [17, 45] using mini-nutritional assessment (MNA). Only one study [54] used Hamilton Depression Scale (HAMD), which showed that the depression degree of acupuncture group was lighter than the control group.

### 3.11. Blind Method Analysis

**Figure 16:**
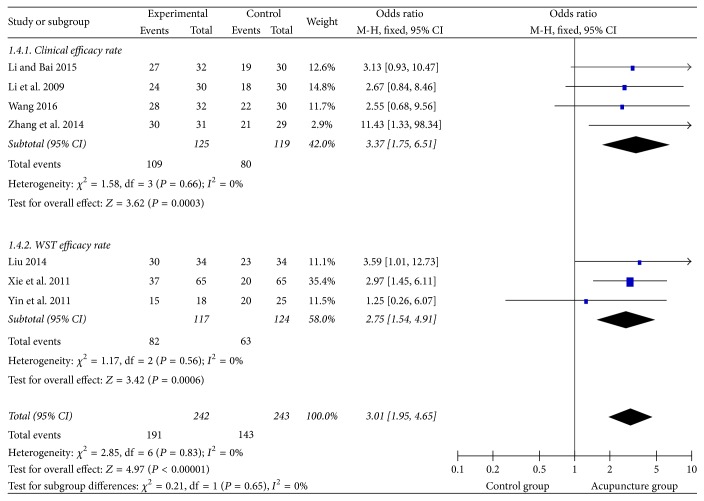
The forest diagram of single blind clinical efficacy rate.

We extracted 7 studies using blind method from the enrolled studies, among which 4 studies used the clinical therapeutic efficiency and 5 used Watian Swallowing Test efficacy rate. There was no heterogeneity (*I*^2^ = 0%) after pooling them with dichotomous data and risk ratio (RR) (Figure 16). So the fixed effect model was used. The rectangle was on the right of equivalent line and the therapeutic efficiency of acupuncture group was 3.01 times as much as the control group. The result indicated that the acupuncture group could improve the therapeutic efficiency of dysphagia after stroke (RR = 3.01, 95% CI: 1.95 4.65, *Z* = 4.97, and *P* < 0.00001).

**Figure 17:**
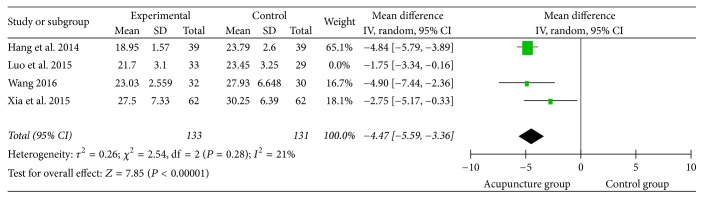
The forest diagram of single blind SSA.

Among the studies employing blind method, 4 of them used the SSA as the assessment indicator with continuous data and mean difference (MD). High heterogeneity was found after combined analysis. Sensitivity analysis revealed that heterogeneity might be due to the use of the test method and the gender imbalance in the clinical cases from one study [17]. The heterogeneity was lower (*I*^2^ = 21%) after removing it. We could see from the figure (Figure 17) that the rectangle was on the left of equivalent line, with a trend that acupuncture group could lower the SSA scores (MD = −4.47, 95% CI: −6.59 −3.36, *Z* = 7.85, and *P* < 0.00001).

### 3.12. Adverse Reactions Report

Only 13 studies mentioned the security index, including how to prevent the subcutaneous hemorrhage, needle sickness, curved needle, broken needle, and the handing method during acupuncture process. Meanwhile, some studies reported the influence caused by the adverse reactions, not the others.

## 4. Discussion

The study indicated that the therapeutic efficacy of acupuncture or acupuncture combined with other intervention was better than the control group, though some pooled results had higher heterogeneity. The interventions such as the acupuncture, rehabilitation training, and swallowing training were related to the professional skill of the practitioners, the same as the efficacy evaluation. Meanwhile, the various source of cases might lead to difference statistic results.

### 4.1. Comparison with Other Literatures

The acupuncture alone or combined with other interventions is widely used for dysphagia after stroke in China. There exists some evidence about the acupuncture for dysphagia after stoke. One report [88] stated although acupuncture had a tendency to improve dysphagia after stroke, it could not get the positive conclusion. There was report [89] which indicated that acupuncture combined with the swallowing rehabilitation training had certain advantage. Long and Wu [90] pointed out that acupuncture may be benefit for dysphagia, but high-quality research was needed. The present study reworked out inclusion and exclusion criteria to evaluate the efficacy of acupuncture for treatment of dysphagia after stroke and showed stronger evidence in the result.

### 4.2. Strengths and Limitations

In this paper, the studies included single blind method pooled to analysis alone and showed stronger evidence on acupuncture for treatment of dysphagia after stroke. We incorporated all researches in the past 5 years. Considering the clinical application of the intervention in this paper was special, such as the feeling of the patient. It was difficult to achieve true double blind. The studies using single blind method achieved the blind method to some extent. There was no or lower heterogeneity after combining.

On the outcome indicator of the dysphagia, most of the studies used the Watian Swallowing Test, SSA, Fujishima Rating Scale, and so on. Only 5 studies [21, 38, 50, 72, 87] used the golden standard-videofluoroscopy (VFSFF) [91] as the assessment indicator. The Watian Swallowing Test was put forward by the Toshio Watian from Japan, which was used as preliminary screening for dysphagia. Meanwhile, it was dependent on the feeling of patients and susceptible to them, which made the inconsistencies with most results in clinical and laboratory inspection [92, 93]. However, it was classified clearly and simply to use. So it was employed in many researches clinically [18, 21, 23]. Therefore, the choice of evaluation criteria needs to be more rigorous and scientific in the clinical trial design. In order to increase the reliability, high level evaluators should be chosen to evaluate the efficacy for dysphagia simultaneously.

However, there were several limitations of this review. Some research used the acupuncture combined with other interventions on the basic of the control group. And it was easily mixed with the effect of the acupuncture. Therefore, for experiment group, acupuncture alone or combined with the interventions of the control group might increase the reliability.

Some studies [94, 95] showed that acupuncture seemed to be safe in the subacute phase of ischemic stroke and cardiac arrhythmia. Others [96] indicated that the safety of acupuncture needs further evidence. And some researches [97, 98] show that the occurrence of the adverse events during acupuncture was closely related to the competency of the practitioners and the safety system of acupuncture. However, in the process of literature retrieval, we found that most of the literatures included in this paper paid too much attention to the validity of acupuncture and ignored the influence of adverse event during acupuncture. Therefore, we should consider the security issues in the research design. The unfinished trials caused by the security issues should be reported perfectly according to international standard [99] to ensure the data's integrity.

## 5. Conclusion

In conclusion, acupuncture for dysphagia after stroke has therapeutic efficacy. And the acupuncture is safe and reliable within a certain range. More strict evaluation standard and high-quality RCT design are necessary for further exploration on the acupuncture for treatment of dysphagia after stroke.

## Figures and Tables

**Table 1:** Retrieved literatures.

Database	CNKI	WanFang	CSJD	CBM	PubMed	EMBASE	Cochrane	SCI
Number	1361	1451	593	388	41	0	2	13

**Table 2:** The basic characteristic of the included studies.

Reference	Simple size T/C (M/F)	Design/blind	Diagnostic criteria	Intervention	Control	Mean age (T/C)	Treatment course	Outcome measures
Luo et al. [17] 2015	33/29	RCT/single blind	FNCOCD-1994	A + BT + RT	BT + RT	68.45 ± 9.73/66.90 ± 11.55	38.82 ± 48.77/27.14 ± 47.30	SSA + MNA
Xia et al. [18] 2015	62 (35/27)/62 (36/26)	RCT/single blind	NIHSS	A + ST	ST	65.3 ± 14.2/66.1 ± 14.3	NR	SSA + DOSS
Xie et al. [19] 2011	70 (39/31)/70 (28/32)	RCT/single blind	FNCOCD-1994	A	BT + RT	66.0 ± 8.4/68.4 ± 9.1	64.7 ± 32.0/60.0 ± 36.0	WST
Yin et al. [20] 2011	18 (10/8)/25 (11/9)	RCT/single blind	FNCOCD-1994	A + ST + ES	ST + ES	69.52 ± 6.01/65.41 ± 7.01	25.60 ± 3.09/24.32 ± 2.78	WST + IFRS
Zhang et al. [21] 2014	29 (9/22)/31 (8/21)	RCT/single blind	RFNCOCD-1994	A + TCM + RT	BT + RT	65.55 ± 7.05/62.21 ± 8.37	42.56 ± 14.26/45.12 ± 12.56	VFSS + WST
Wang [22] 2016	30 (18/14)/30 (17/13)	RCT/single blind	RFNCOCD-1994	A + BT + ES	BT + RT	63.81 ± 8.445/64.87 ± 9.228	43.69 ± 18.39/42.0 ± 18.134	WST + SSA + SWAL-QOL
Li and Bai [23] 2015	32 (22/10)/30 (20/10)	RCT/single blind	RFNCOCD-1994	A + BT + RT	BT + RT	55.17 ± 4.73/54.97 ± 5.46	NR	WST
Liu [24] 2014	34/34	RCT/single blind	GFDTAISC-2010	A + BT + RT	BT	61.65 ± 8.253/63.71 ± 7.112	4.56 ± 4.294/4.18 ± 4.159	WST
Hang et al. [25] 2014	39 (16/23)/39 (19/20)	RCT/single blind	RFNCOCD-1994	A + BT + OPT	BT	56.36 ± 10.55/56.36 ± 10.55	17.36 ± 15.52/19.49 ± 16.91	SSA
Li et al. [26] 2009	30/30 NR	RCT/single blind	FNCOCD-1995	A + RT	RT	NR	NR	SSA
Zhu et al. [27] 2012	82 (59/23)/82 (53/29)	RCT/no blind	FNCOCD-1995	A + SUG	SUG	60.8/62.4	NR	WST + NIHSS + Barthel
Chen and Zhang [28] 2016	30 (16/14)/30 (20/10)	RCT/no blind	CCDPATGBNBCMA-2007	A + RT	RT	61.63 ± 10.87/60.90 ± 10.53	47.68 d/41.63 d	WST
Chen et al. [29] 2014	168 (117/5)/171 (131/4)	RCT/no blind	SOSNCOCD-2004	A + RT + BT	BT + RT	64.40 ± 11.20/64.05 ± 11.35	35.29 ± 32.84/33.12 ± 29.12	WST + TIFRS
Wang [30] 2013	30 (16/14)/30 (15/15)	RCT/no blind	FNCOCD-1996	A + BT + TCM	BT	65.3 ± 8.2/66.4 ± 5.4	14.98 ± 13.02/15.79 ± 13.76	SSA
Duan [31] 2014	40 (24/16)/40 (25/15)	RCT/no blind	FNCOCD-1994	A + SM + BT	BT	52.5 ± 3.7/52.5 ± 3.7	NR	WST
Bao and Zou [32] 2015	30 (21/9)/30 (22/8)	RCT/no blind	FNCOCD-1997	A + WM	WM	68.40 ± 7.166/68.77 ± 6.606	48.80 ± 23.57/50.03 ± 24.33	WST
Fan et al. [33] 2007	30 (13/17)/30 (14/16)	RCT/no blind	RFNCOCD-1996	A + WM	WM	67.76 ± 4.34/68.03 ± 4.05	46.37 ± 25.34/47.18 ± 26.15	WST

Feng et al. [34] 2016	30 (18/12)/30 (19/11)	RCT/no blind	GFDTAISC-2010	A + RT + BT	BT + RT	60 ± 12/58 ± 22	38 ± 18/39 ± 18	WST + VFSS
Zhen et al. [35] 2012	30 (16/14) 30 (13/17)	RCT/no blind	FNCOCD-1996	A + BT	BT	61 ± 3/60 ± 3	7 d–10 m/6 d–10 m	WST
Chang et al. [36] 2014	38 (27/11)/36 (28/8)	RCT/no blind	RFNCOCD-1996	A + ES + BT	ES + BT	46 ± 10/44 ± 11	16.6 ± 4.8/17.3 ± 5.2	IFRS
Wu et al. [37] 2011	80 (54/26)/75 (52/23)	RCT/no blind	TEESTCMDFS-1996	A + BT + ST	BT + ST	68/66	NR	WST
Zhang and Yin [38] 2012	62 (32/30)/56 (22/34)	RCT/no blind	RFNCOCD-1995	A + BT + ST	BT + ST	70 ± 2/68 ± 2	30.86 ± 12.72/31.78 ± 11.23	MBSAS + WST + DRS
Chen [39] 2008	30 (19/11)/30 (17/13)	RCT/no blind	TEESTCMDFS-1997	A + BT	BT	42–79/40–81	NR	WST
Mao et al. [40] 2015	40 (22/18)/40 (21/19)	RCT/no blind	FNCOCD-1995	A + RT + BT	RT + BT + SDTA	63.64/62.9	56.2 ± 7.239/54.8 ± 6.033	VFSS
Guo and Li [41] 2016	40 (23/17)/40 (25/15)	RCT/no blind	FNCOCD-1997	A + BT	BT + ST	55.28 ± 10.34/56.12 ± 11.47	21.19 ± 8.28/20.49 ± 9.15	SSA
Gao et al. [42] 2016	40 (24/16)/40 (22/18)	RCT/no blind	FNCOCD-1994	A + ST	ST	57.8 ± 4.9/58.2 ± 5.1	NR	WST
Bai [43] 2016	40 (16/24)/40 (15/25)	RCT/no blind	FNCOCD-1994	A + SDTA	SDTA	63.34 ± 9.04/63.15 ± 9.24	NR	WST
Song [44] 2012	30 (19/11)/30 (20/10)	RCT/no blind	CCDPATGBNBCMAG-2005	A + ST + BT	ST + BT	61.3/61.52	2.26 m/2.09 m	WST + IFRS
Zhou et al. [45] 2014	30 (19/11)/30 (20/10)	RCT/no blind	CCDPATGBNBCMA-2005	A	RT	65.63 ± 9.33/64.35 ± 8.26	NR	WST + MNA + Barthel
Liu [46] 2014	45 (28/17)/42 (27/15)	RCT/no blind	FNCOCD-1996	A + ST	ST	52.3 ± 8.7/53.6 ± 8.5	NR	WST
Li et al. [47] 2013	30 (20/10)/30 (21/9)	RCT/no blind	FNCOCD-1996	A + BT + ST	BT	56.9 ± 4.6/57.1 ± 3.7	NR	WST
Wang [48] 2011	30 (18/12)/30 (16/14)	RCT/no blind	FNCOCD-1995	A + BT	BT	56.5/56.8	6–35 d/7–34 d	WST
Liu and Zheng [49] 2014	33 (17/16)/31 (14/17)	RCT/no blind	FNCOCD-1996	A + BT	BT	61.7/59.8	NR	WST
Gu [50] 2011	35 (22/13)/35 (23/12)	RCT/no blind	FNCOCD-1996	A + RT + BT	BT + RT	71.98 ± 10.19/70.74 ± 11.58	14.69 ± 15.76/17.11 ± 15.52	CT7R
Gao et al. [51] 2014	52 (31/21)/49 (27/22)	RCT/no blind	CCDPATGBNBCMA-2007	A + BT + SDTA	BT + SDTA	60.25 ± 8.36/61.37 ± 7.36	NR	WST
Chen [52] 2016	30 (17/13)/30 (18/12)	RCT/no blind	FNCOCD-1995	A + BT + RT	BT + RT	62.90 ± 10.04/63 ± 9.83	NR	VFSS + Rosenbek
Ruan et al. [53] 2015	25 (12/13)/25 (14/11)	RCT/no blind	CCDPATGBNBCMA-2005	A + BT + ST	BT + ST	58.01 ± 10.74/57.98 ± 11.82	47.02 ± 7.47/46.87 ± 6.96	IFRS

Wang [54] 2015	46 (35/11)/45 (31/14)	RCT/no blind	FNCOCD-1997	A + BT	RT + BT	61 ± 10/64 ± 10	54.63 ± 27.18/51.93 ± 23.10	WST + SSA + SWAL-QOL + HAMD + MBI
Li et al. [55] 2015	65 (47/18)/65 (49/16)	RCT/no blind	FNCOCD-1996	A + BT	BT	63.87 ± 5.24/63.96 ± 5.33	2.32 ± 1.79/3.38 ± 1.90	WST + IFRS
Zhang et al. [56] 2011	30/30	RCT/no blind	FNCOCD-1995	A + ST	ST	NR	NR	WST
Qu [57] 2009	30 (17/13)/30 (18/12)	RCT/no blind	FNCOCD-1997	A + RT	RT	69.06 ± 6.67/66.84 ± 10.39	27.27 ± 13.76/27.30 ± 8.11	WST
Chen and Lin [58] 2016	60 (38/22)/60 (41/19)	RCT/no blind	RFNCOCD-1996	A + RT	RT	65/63	9–40 d/10–38 d	WST
Gao and Zhu [59] 2015	30/30 (30/30)	RCT/no blind	FNCOCD-1996	A + BT	BT	45–75	5–45 d	WST
Liu et al. [60] 2012	36 (22/14)/36 (25/11)	RCT/no blind	FNCOCD-1994	A + BT	BT	57.6 ± 8.2/58.5 ± 8.7	35.4 ± 6.5/34.8 ± 7.1	WST
Dong et al. [61] 2014	30 (16/14)/30 (17/13)	RCT/no blind	FNCOCD-1995	A + RT	RT	63/62	35/36	WST
Zhang [62] 2014	50 (28/22)/46 (26/20)	RCT/no blind	FNCOCD-1996	A + BT + NEST	RB + BT	67.5 ± 7.2/68.2 ± 6.4	8.8 ± 1.2/9.6 ± 1.4	WST
Li et al. [63] 2014	20 (11/9)/20 (12/8)	RCT/no blind	FNCOCD-1995	A + BT + ST +	BT + ST	60.4 ± 4.6/61.3 ± 4.2	15.3 ± 2.7/14.7 ± 2.1	WST + OFS
Wang and Yang [64] 2013	45 (20/25)/45 (21/24)	RCT/no blind	FNCOCD-1995	A + ST + BT	ST + BT	61.33 ± 4.19/61.61 ± 5.34	29.12 ± 7.09/31.41 ± 6.32	WST
Ding and Zhang [65] 2013	30 (17/13)/30 (19/11)	RCT/no blind	FNCOCD-1994	A + BT	BT	63.14 ± 7.28/62.47 ± 6.91	27.2 ± 7.5/28.6 ± 7.9	WST
Fang [66] 2014	30 (16/14)/30 (15/15)	RCT/no blind	FNCOCD-1996	A + BT + CMPPS	BT + ST	52.8 ± 6.9/53.9 ± 6.0	34.1 ± 15.3/31.4 ± 12.6	WST
Qin [67] 2015	30 (26/4)/30 (21/9)	RCT/no blind	RFNCOCD-1995	A + BT	RT + BT	61.42 ± 13.65/63.86 ± 10.83	34.99 ± 8.75/31.18 ± 7.21	WST + SSA + ADL
S. Zhang and G. M. Zhang [68] 2014	87 (64/23)/87 (58/29)	RCT/no blind	CCDPATGBNBCMA-2007	A + RT	RT	63.86 ± 10.55/64.61 ± 9.70	28.45 ± 23.42/31.48 ± 27.80	WST + IFRS
Zhu and Zhao [69] 2015	30 (18/12)/30 (16/14)	RCT/no blind	TEESTCMDFS-1996	A + BT	RT	53.60 ± 12.96/56.10 ± 10.81	6.83 ± 1.60/7.05 ± 1.33	WST
Fu [70] 2016	53 (30/23)/48 (28/20)	RCT/no blind	FNCOCD-2007	A + RT + BT	RT + BT	52.8 ± 10.4/55.4 ± 13.8	6.8 ± 2.3/8.5 ± 3.1	SSA + SWAL-QOL
Zhang et al. [71] 2007	110 (67/43)/110 (62/48)	RCT/no blind	RFNCOCD-1995	A + RT	RT	53.16 ± 6.84/51.37 ± 8.63	NR	WST
Wei and An [72] 2012	50 (34/16)/50 (36/14)	RCT/no blind	CCDPATGBNBCMA-2005	A + BT + RT	BT	57.8/57.7	3–45/4–46	WST
Yin [73] 2013	57 (32/25)/56 (30/26)	RCT/no blind	RFNCOCD-1996	A + RT + BT	RT + BT	62.5 ± 6.5/60.8 ± 7.4	11.5 ± 2.2/10.3 ± 1.3	SSA + SWAL-QOL

Huang et al. [74] 2011	28 (16/12)/28 (15/13)	RCT/no blind	FNCOCD-1995	A + RT	RT	38–75/38–73	15 d–6 m/18 d/5 m	WST
Chen et al. [75] 2011	28 (12/16)/28 (13/15)	RCT/no blind	FNCOCD-1995	A + ST + NEST	ST	57.71 ± 9.17/59.50 ± 8.79	50.21 ± 21.59/49.14 ± 20.76	WST
Wang and Cui [76] 2011	70 (49/12)/70 (52/18)	RCT/no blind	FNCOCD-1996	A + LFPT + BT	BT	66.12/64.87	65.4/71.1	WST
Li [77] 2016	36 (23/13)/36 (24/12)	RCT/no blind	FNCOCD-1997	A + BT + AM	BT	52.98 ± 4.93/53.61 ± 4.81	13.65 ± 5.25/12.53 ± 6.16	WST
Yi [78] 2014	30 (19/11)/30 (18/12)	RCT/no blind	CCDPATGBNBCMA-2010	A + SUMM	SUMM	62.03 ± 10.14/63.90 ± 8.64	66.23 ± 47.94/70.23 ± 44.36	SSA + ADL
Jia et al. [79] 2014	30 (23/7)/30 (21/9)	RCT/no blind	FNCOCD-1995	A + RT	RT	58.30 ± 7.87/56.47 ± 8.43	<6 M	WST
Huang et al. [80] 2013	30 (17/13)/30 (16/14)	RCT/no blind	FNCOCD-1995	A	TCM	65 ± 3/67 ± 2	109 ± 4/108/9	WST
Feng and Sun 2016 [81]	30/30	RCT/no blind	FNCOCD-1996	A	BT	NR	NR	WST
Chen et al. [82] 2015	30/30	RCT/no blind	FNCOCD 1995	A + BT + ST	BT + ST	NR	NR	IFRS
Feng et al. [83] 2015	45 (32/13)/45 (29/16)	RCT/no blind	FNCOCD-1995	A + BT + LFES	LFES + BT	63.12/52.36	56.52/45.12	WST
Yu and Hu [84] 2012	40 (23/17)/38 (20/18)	RCT/no blind	FNCOCD-1995	A + RT + BT	RT + BT	62.51 ± 10.17/62.51 ± 10.17	13.78 ± 2.62/14.56 ± 2.48	WST
Zhao and Zhang [85] 2012	94 (48/46)/92 (47/45)	RCT/no blind	FNCOCD-1995	A + STA	STA	59.78/60.03	NR	WST
Yu et al. [86] 2012	30 (19/11)/30 (13/17)	RCT/no blind	FNCOCD-1995	A + BT	BT	54.30 ± 11.5/55.50 ± 10.7	30.25 ± 10.53/31.12 ± 8.92	WST
Zheng [87] 2014	30 (21/9)/30 (23/7)	RCT/no blind	FNCOCD-1994	A + ST	ST	68.3 ± 13.84/70.26 ± 11.97	43.37 ± 24.37/44.30 ± 22.52	CT7R + SSA + VFSS + WST

(1) Diagnosis: FNCOCD, the diagnostic criterion of the Fourth National Conference on cerebrovascular diseases in 1994 or the revised diagnostic criterion in 1995 or 1996; FNCOCD the revised diagnostic criterion in 1995 or 1996; CCDPATGBNBCMA, the “Chinese Cerebrovascular Disease Prevention And Treatment Guidelines (Try Out)” established by Neurology Branch of Chinese Medical Association according to the 2005 or 2007 Disease Control Division; GFDTAISC2010, the guidelines for diagnosis and treatment of acute ischemic stroke in China 2010 Edition; NIHSS, National Institutes of Health Stroke Scale; TEESTCMDFS, the therapeutic effect evaluation standard of TCM diagnosis for stroke; SOSNCOCD, summary of the Sixth National Conference on cerebrovascular diseases. (2) Measures: CT7R, Caiteng 7 Rank; MBSAT, medical bedside swallowing assessment scale; DRS, dysphagia rank scale; WST, Watian Swallowing Test; SSA, standard swallowing assessment; VFSS, videofluoroscopy; IFRS, Ichiro Fujishima Rating Scale; ADL, Activity of Daily Life; WALQOL, Swallowing-Related Quality of Life; DSS, dysphagia severity scale; OFS, oral function score. (3) Intervention: SM, swallowing mixture; A, acupuncture; RT, rehabilitation; BT, basic treatment; SUMM, stroke unit management model; ST, swallowing treatment; SUG, stroke unit group; ES, electrical stimulation; TCM, traditional Chinese medicine; WM, Western medicine; OPT, oral positioning therapy; AM, acupoint massage; NEST, neuromuscular electrical stimulation; MS, muscle electrical stimulation; LPT, low frequency pulse electrotherapy; ES, electrical stimulation; SDTA, swallowing disorder therapeutic apparatus; CMPPS, cold medicine Popsicle pharyngeal stimulation; IS, ice stimulation; NR, no report.

## Abbreviations

NIHSS: National Institutes of Health Stroke Scale
CT: Computerized tomographic scanning
MRI: Magnetic resonance imaging
SCI: Science Citation Index
CNKI: Chinese National Knowledge Infrastructure
CSJD: Chinese Scientific Journals Database
CBM: Chinese Biomedical Literature Database
CT7R: Caiteng 7 Rank
WST: Watian Swallowing Test
VFSS: Videofluoroscopy
ADL: Activity of Daily Life
IFRS: Ichiro Fujishima Rating Scale
WALQOL: Swallowing-Related Quality of Life.
